# Managing flood disasters on the built environment in the rural communities of Zimbabwe: Lessons learnt

**DOI:** 10.4102/jamba.v10i1.542

**Published:** 2018-05-30

**Authors:** Ernest Dube, Oliver Mtapuri, Jephias Matunhu

**Affiliations:** 1 Department of Development Studies, Midlands State University, Zimbabwe; 2 School of Built Environment and Development Studies, University of KwaZulu-Natal, South Africa

## Abstract

This article is about managing flood disasters affecting the built environment in the rural communities of Zimbabwe. Using Tsholotsho district in Matabeleland North province as a case study, the authors argue that flooding has adversely impacted the built environment through destroying infrastructure. The principal objectives of this study were to establish the impact of flood disasters on the built environment, to demarcate factors that perpetuate communities’ vulnerabilities to flooding and to delineate challenges that negate the management of flood disasters in the built environment. This qualitative study was based on a purposive sample of 40 participants. Data were collected through semi-structured interviews and observation methods. The findings were that floods can damage human shelter, roads, bridges and dams. Locating homesteads near rivers and dams, using poor-quality construction materials, and lack of flood warning were found to perpetuate vulnerability to flooding. Poverty and costs of rebuilding infrastructure, lack of cooperation between the communities and duty-bearers, and failure to use indigenous knowledge were found to be impeding the management of flood disasters. The study concluded that flood disasters can wipe out community development gains accumulated over many years. Further, community vulnerability to flooding in the built environment is socially constructed. The study posits that addressing the root causes, reducing flood vulnerability and avoiding risk creation are viable options to development in the built environment. Lastly, reconstruction following flood disasters is arduous and gruelling, and not an easy exercise.

## Introduction

Flood disasters have a propensity to impact the built environment and cause huge losses through destroying property, including buildings and infrastructure (Cigler 2017; Price & Viginovic 2008). Evidence shows that flood disasters can destroy the built environment, thereby creating a fresh start in addressing previous construction imperfections. Flood disasters can wipe out the built environment, leaving affected communities with difficulties to recover on their own without external support. Delaney and Shrader (2000) observe that disasters are largely intractable problems that test resilience of communities to effectively protect their populations and infrastructure. Flood disasters are also caused by failed infrastructural development. Therefore, flood disaster management has remained an issue of concern in modern societies. For instance, when flood disasters impact homes, shelter support and infrastructure restoration may become a daunting task (Fu, Lin & Shieh 2013). As such projects demand quick restoration of affected communities, mishandling of projects can exacerbate existing community vulnerabilities (Johnson, Lizarralde & Davidson 2006). However, with proper flood management, impact of disasters can be minimised.

This study focuses on managing flood disasters on the built environment in Tsholotsho district, south-west of Zimbabwe. The principal objectives of the study are threefold, namely, to establish the impact of flood disasters on the built environment, to demarcate factors that perpetuate vulnerability to flooding and to delineate the challenges that negate the management of flood disasters impacting the built environment.

### Statement of the problem

The management of flood disasters in Tsholotsho district in the Matabeleland North province, south-west of Zimbabwe, has not been effective enough to save the built environment. Of late, the district has been inundated by destructive flooding events. The floods have severely impacted the built environment, damaging human shelter among other forms of infrastructure. Communities most affected have been those located along the Gwayi, Zombani and Manzamnyama rivers, and communities settled near Gariya and Bhudani dams. The Government of Zimbabwe has at times tried to restore the damaged shelter and infrastructure through some reconstruction processes. However, following subsequent severe flooding, the built environment has always experienced the same impact of being destroyed by the forceful floods. As such, it is imperative that the government comes up with new robust measures that can save the built environment. Such measures have a potential to benefit the communities through minimising the impact of future flooding events. If no meaningful measures are taken by the duty-bearers to deal with the impact of flooding on the built environment in the district, the communities will continue to suffer huge losses. Such losses may even be permanent, considering that many communities in rural districts are poor, with little or no means to recover from disaster impact (Cigler 2017; Price & Vijonivic 2008).

## Review of related literature

This section of the article reviews the literature relevant to the study. The literature is interrogated and discussed in a manner that agrees with the study’s objectives. The first objective focuses on the impact of flood disasters on the built environment.

### Flood disaster impact on the built environment

Globally, flood disasters of various magnitudes have continued to severely impact the built environment. According to Lindell (2013), disaster damage on the built environment is twofold, that is, damage to structures and damage to the contents of the structures. Collins and Simpson (2007) also note that from 1971 to 1995, of the 1.5 billion people affected by floods worldwide, more than 81 million people were left homeless through infrastructure destruction. Every community is at the risk of flood disasters, although the level of risk may differ depending on the nature of physical structures, hazards and social variables that constitute the built environment. Ariyabandu and Wickramasinghe (2003) assert that a community’s level of risk to flood disasters is determined by the magnitude of the flood hazard, and the community’s calculation of the level of vulnerability. Henderson (2004) added that the level of disaster risk and vulnerability in developing countries is attributed to socio-economic stress, ageing and lack of physical infrastructure. As such, floods often result in huge numbers of tangible losses in the built environment of developing and less developed countries. Such losses may include human, material, economic and cultural losses. Cultural losses entail damage to cultural and historic buildings, and places of worship (Environmental Planning Collaborative & TCG International 2004). Therefore, impact of flood disasters on the built environments suggests that duty-bearers have a lot of work. There is a need for governments to reconsider more meaningful and sustainable measures for dealing with flood disasters affecting the built environment. The next subsection looks at the factors that contribute to flood vulnerability in the built environment.

### Factors that perpetuate flood vulnerability in the built environment

Certain factors may perpetuate communities’ vulnerability to flooding, leading to disaster occurrence in the built environment. This study chose the three common aspects of flood vulnerability, namely, use of poor building materials and communities’ failure to adhere to building codes, sociological aspects of communities and cultural beliefs. These factors were chosen because they were believed to be the major drivers contributing to flood disasters in modern societies. Mudavanhu (2014) posits that factors such as terrain, poor physical structures, lack of building codes for standard buildings and lack of resources have been understood to influence flood vulnerability in the built environment. In order to avert losses associated with flooding, these factors need to be thoroughly addressed by the disaster risk reduction duty-bearers.

#### Poorly built structures and non-conformity to building codes

Previous studies on disaster risk reduction have shown that structures constructed using poor, non-durable construction materials are vulnerable to flood hazards. Therefore, players in the construction industry are encouraged to carefully choose building materials when constructing structures. Substandard building practices are understood to aggravate flood disaster impact on the built environment. Deficiencies, which manifest in the failure to enforce building codes, if not addressed, may prevail even in the post-flood disaster reconstruction period. Vulnerability to flooding in the built environment can, therefore, be reduced through the building of resilient structures, through regulating social behaviours, and through the provision of adequate early warnings, among other strategies (Pelling 2003). Appropriate enforcement of building codes may limit the number of casualties and minimise losses created by flooding. For instance, on 13 July 1995, heavy rain pounded Senirkent in Turkey, creating a severe flood that destroyed 320 dwellings, of which 195 structures were completely destroyed, 18 moderately damaged and 107 slightly damaged (Ozden 2004). According to Ozden (2004), the destroyed dwellings were constructed using mud-brick, a weak material that could not resist the flood forces. It is possible that proper building codes were not followed in the construction of the structures. If properly enforced, building codes are an important component towards reducing flood losses.

Satterthwaite et al. (2007) note that in developing countries, some communities do not have suitable roads, people live in poor-quality homes and at times on illegally occupied land. This can inhibit investment to build more resilient structures, as no meaningful development can take place on illegally occupied land. As people settled in such areas usually live under the fear of being forcefully evacuated, they tend to build temporary, weak structures. Such infrastructure can expose communities to future flooding. Also, a point to note is that many residents in developing countries are tenants whose landlords have no capacity to invest in better-quality buildings (Satterthwaite et al. 2007).

#### Social configurations of communities

Social setting of communities is another factor that can influence flood disasters within built environments. Sociological aspects and configurations of communities can worsen some people’s vulnerabilities and fuel the intensity of flooding events (Velasquez & Tanhueco 2005). Therefore, it is believed that calamitous flooding events can emanate from extremes in geophysical processes. For example, flooding in the built environment may occur owing to housing structures located on marginal sites and the physical characteristics of an area (Oelofse 2002). Areas of high risk that may influence flood disasters include floodplains, coastal hurricane areas, among others. The authors contend that when a flood disaster strikes, even the most durable structures may be impacted if such structures are located in high-risk areas. Duty-bearers should, therefore, consider situating structures in places that are not prone to flooding.

Social arrangements of communities have also been understood to be influenced by poverty which may cause poor households to settle in places prone to flooding. People from poor households often have higher physical vulnerabilities because they live in weaker structures that follow older, less stringent building codes, in addition to inferior quality construction materials. Therefore, the poor are more vulnerable to disasters, and they are sometimes forced to accept, as permanent shelter, what originally would have been intended to be temporary housing (Lo & Oreta 2010). Shah, Khan and Qazi (2013) observe that the poor are forced to remain in risky temporary housing structures even after the rich households have relocated to permanent housing. In the reconstruction phase, it would be ideal to settle communities away from flood-prone places as a way of reducing future vulnerabilities. McEntire (2004) supports this idea and states that social changes would have to take place if future flood losses were to be minimised.

#### Cultural beliefs

Cultural beliefs can also determine the impact of floods on the built environment, creating unnecessary disaster losses. Mileti et al. (1995:122) observe that ‘elements of culture… constrain effective and sustainable adaptation to natural hazards’, and floods are no exception. Therefore, alterations in people’s cultural beliefs and behaviour may be a means to reducing the impact of flood hazards (Mileti et al. 1995), as some people prefer to remain in flood-prone areas owing to cultural reasons. For instance, some people may not want to move away from mountains or graves of their ancestors because of cultural beliefs that such action would anger the spirits. However, the authors argue that people’s cultural beliefs are not reason enough to settle in flood-prone zones. Such a move is discouraged as it perpetuates people’s vulnerability to flood hazards and resultantly leading to flood disaster losses.

To some communities, natural disasters such as floods are an ‘act of God’ and, therefore, little can be done by human beings about disasters. Such communities usually undertake negligent and dangerous manoeuvres, for instance, building residential structures in disaster-prone areas with the hope that as disasters are an act of God, God would protect them from harm. However, available evidence shows that such moves have not helped many communities. Instead, it has been proved that bad cultural practices, as well as weak disaster risk management and development institutions, augment the vulnerability of societies (McEntire 2003). Bad human cultural practices, therefore, play a major part as they contribute to creating disasters. In that way, flood disasters are also a reflection of ‘the ongoing social order… and the larger historical circumstances that shape or frustrate these matters’ (Hewitt 1983:25). The next section reviews the literature to expose challenges that impede the reconstruction process. The authors argue that duty-bearers should be mindful of such challenges in order to effectively serve flood-impacted communities.

### Challenges to reconstruction in the built environment after flood impact

Although the reconstruction phase following flood impact can provide development with a new meaning, the process can be faced with serious challenges. It has often been observed that in comparison with pre-disaster project construction, post-disaster reconstruction environment is often chaotic, dynamic and complex (Birkland 2006; Davidson et al. 2007). As such, duty-bearers should consider several elements in the reconstruction phase, following flood disasters. They may also consider the wider political and social contexts, and operational requirements (Harvey 2005), to complement the expectations and preferences of disaster victims. Provision of resources can also play a major role in the reconstruction process. Previous disaster studies have proved that the reconstruction phase is sometimes dictated by availability of resources, in addition to people’s experiences and capacities (Zuo & Wilkinson 2008). Unwillingness to participate by affected communities and lack of relevant skills are other factors that might impede the success of reconstruction in the built environment. However, Davidson et al. (2007) observe that not all forms of participation would guarantee the best deployment of people’s capabilities. Community members without motivation and necessary skills would hardly achieve programme goals even if there were high levels of participation.

Reconstruction processes after flood impact may also fail owing to shunning of modern technology by construction agents and communities. For example, during the reconstruction following the Indian Ocean tsunami flooding, the Government of India encouraged the use of environment-friendly, low-cost and seismically resistant materials in the flood-impacted areas as part of embracing new technology (Barenstein & Pittet 2007; Steinberg 2007). However, the new construction methods and materials brought by new technology were not popular with some locals and, therefore, were not wholly accepted (Boen & Jigyasu 2005; Schilderman 2004). As a result, the wide use and application of the new technology in areas affected by the floods were very minimal. Benson and Clay (2004) add that there is always some difficulty in implementing new technology, following disaster impact owing to lack of time and financial capacity to drive development projects. Therefore, reconstruction in the built environment to address flood impact should be supported with enough time and funding. Many communities in less developed countries face challenges of securing the necessary construction materials owing to the costs involved. However, if all these challenges are dealt with effectively, reconstruction after flooding can yield positive results. Reconstruction of flood-damaged infrastructure, when guided by best practices, can provide resilience against future threats.

### Towards the ‘build-back-better’ principle

The ‘build-back-better’ principle is an important component of development, which can be enforced in the reconstruction phase following disaster impact. This principle is about the building of better and more improved infrastructure, compared to the infrastructure damaged through a disaster event. We prefer to define the build-back-better principle as ‘positive reconstruction’ because its aim is to address previous construction irregularities such as poor site planning, use of unsuitable building materials and failure to adhere to rules and regulations in the built environment. Such issues can be corrected through improved reconstruction of housing and infrastructure. In addition, the authors argue that the build-back-better principle should be supported with community development principles of simplicity, lifelong learning and empowerment, among other principles, for it to be successful. The principle of simplicity says, simplify things for communities so that they understand, while the principle of lifelong learning is integrated into all aspects of activities to build and support personal skills, knowledge, abilities and resilience of people (ANHLC n.d.). The principle of empowerment respects, values and enhances people’s ability to have control over their lives and encourages people to meet their needs and aspirations in a self-aware and informed way which recognises their skills, experience and potential (ANHLC n.d). According to Mannakkara, Wilkinson and Potangaroa (2014), the build-back-better concept signals an opportunity to decrease the vulnerability of communities to future hazards. This article focuses on the impact of flood disasters on the built environment, and how lessons and experiences drawn from previous flooding events can be an impetus for future development. Therefore, post-disaster reconstruction can be used as an opportunity to build-back-better, through providing better and improved housing and infrastructure (Mannakkara et al. 2014). The common adage that regards experience as the best teacher comes to the fore, when considering rebuilding after a severe flood disaster. Previous disaster experiences and lessons learnt by duty-bearers may act as a better intervention to deal with future flood hazards and disasters. It, therefore, means that despite their negative effects on communities, flood disasters can also bring something positive through lessons learnt. The build-back-better concept mainly focuses on reconstruction, which is the rebuilding and restoring of infrastructure damaged by floods. Such a phase involves helping to restore basic infrastructure and services so that people can enjoy the pattern of life which they had before disaster impact (Davis 2005). Reconstruction restores basic infrastructure and services, and it is also a period to build-back-better through offering improved infrastructure. However, the fact that flood disasters can bring something positive does not mean that disasters should be condoned in societies. Disasters should not be tolerated as they are destructive and can set back development. Flood disasters can reverse development gains accumulated over many years. As such, they are an unwanted visitor in societies.

The authors of this article further argue that the build-back-better concept is a positive move as it offers development with a new meaning. This is so because despite being a painful and long period, rebuilding of damaged infrastructure after flooding may be considered as providing new positive things to the affected communities. Reconstruction is, therefore, understood as a rebuilding measure which involves not only constructing physical structures but also building confidence, self-respect, self-esteem, self-dependency, mutual support and mutual trust of the affected communities (Thurairajah, Amaratunga & Haigh 2008). Manyena (2009) asserts that this phase can provide ‘new things’ such as the construction of schools, health facilities and housing. Such structures do benefit from improved quality, new knowledge and being more hazard resistant. Baradhan (2006) observes that post-disaster reconstruction is the interaction of complex social, technological and economic factors and actions. Therefore, the build-back-better principle provides new technology, improves economy and enhances peoples’ standards of living. However, it should be emphasised that, despite all its benefits, this principle should be embraced as a last resort to address flood disaster impact after all pre-disaster measures have failed. Da Silva (2009) contends that build-back-better introduces improved building practices, new materials and technologies. Therefore, rebuilding after a flood disaster can provide significant opportunities to initiate development programmes (Stephenson 1994). However, despite the major role that the build-back-better principle can play in addressing disaster losses, governments and practitioners should put more focus on the pre-disaster risk reduction phase, instead of being reactive as the idea is to avoid getting into post-disaster interventions. Building back better should only come in after pre-disaster interventions have proved futile. Therefore, it is vital for governments as duty-bearers, and organisations responsible for managing disasters, to consider embracing this principle as an intervention. Just like in the field of health, prevention is better than cure.

However, reconstruction is too big a task for duty-bearers to undertake all by themselves (Environmental Planning Collaborative & TCG International 2004) in order to build-back-better. There is a need to allow populations affected by flood disasters to actively participate in reconstruction efforts so that meaningful development in the built environment is realised. When carrying out post-disaster reconstruction for the built environment in developing countries, such as Zimbabwe, it is encouraged to adopt a community participatory approach, which is owner-driven as it involves members of communities. According to Dube (2015:10), ‘it is important to involve beneficiaries in development initiatives, including disaster risk reduction programmes, in order to enhance programme ownership’.

## Research method and design

### Study area

**FIGURE 1:**
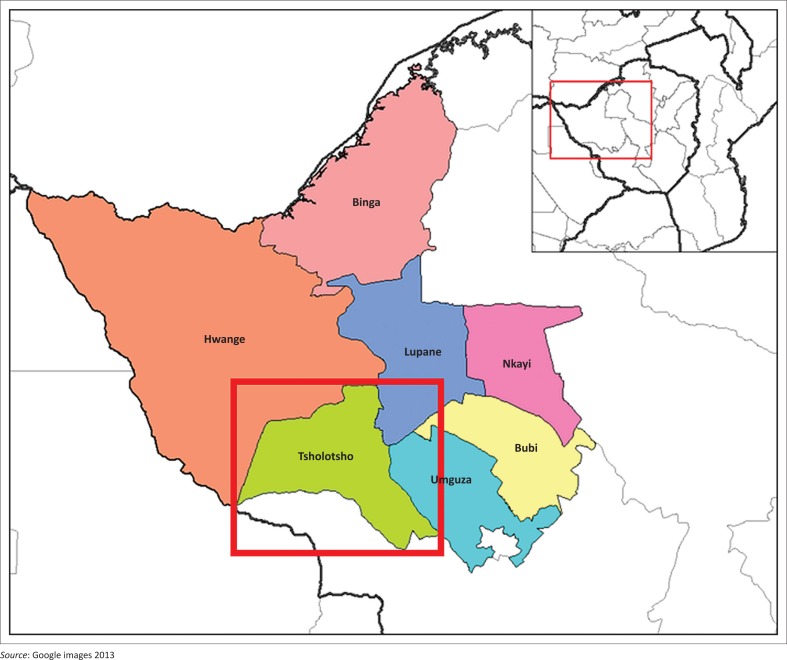
The position of Tsholotsho district on the Map of Zimbabwe.

Tsholotsho district is located in the Matabeleland North province, south-west of Zimbabwe. Many communities in the district are living with flood risk and vulnerability as they live in flood-prone areas along the Gwayi, Zombani and Manzamnyama rivers, while others have settlements situated in floodplains and low-lying areas (Dube 2017). Although the district has a total of 22 wards, the study focused on Wards 5, 6 and 8, which are considered the most flood-prone in the district. The common types of shelter in the district are traditional huts constructed from mud, pole and thatch. The infrastructure consists of many gravel roads, and one tarred road linking the district with Lupane district and Bulawayo. There are also bridges, dams, school buildings, church buildings and business premises. Although the district is categorised as a dry area and lies in the ecological zone of Region 5, high levels of flooding have been experienced of late owing to climate variability and increased rainfall. The problem of flooding started to intensify around year 2000, with the floods of 2000, 2001, 2013, 2014 and 2017 being significant events which disrupted the communities and caused huge losses (Dube 2017). Indicated below (Figure 1) is the map showing the position of Tsholotsho district in Zimbabwe.

As can be observed from the map, Tsholotsho is one of the seven districts that make up Matabeleland North province. The other districts in the province are Hwange, Binga, Lupane, Nkayi, Bubi and Umguza districts (Figure 1). According to Zimstat (2012), Tsholotsho district has an estimated population of 115 119 people based on the 2012 national census.

### Procedure

This study was qualitative, phenomenological and interpretive. The study sought to learn from the participants’ lived experiences of being affected by the flood phenomenon in their built environment. Purposive sampling was adopted and 40 members of the community participated. The participants were deliberately chosen on the understanding that they had experienced flood impact in the past and, therefore, were information-rich and suitable for the study. Data were collected through semi-structured interviews and observation method. These two methods provided relevant and detailed qualitative information. The semi-structured interviews allowed the participants to give information without restrictions, while the observation method afforded the researchers an opportunity to gather onsite first-hand information. Structures damaged by the floods and the construction materials used were observed by the researchers. The participants were the gatekeepers and people whose structures were damaged by previous flood disasters. A thematic analysis was used to analyse the data, with the research themes being derived from literature review so that they resonate with the objectives of the study. The large volumes of raw data collected were analysed to obtain usable and useful information. According to Patton (2002), the purpose of analysing data is to transform data into findings. In line with the study’s qualitative approach, data analysis was performed in order to make sense of the research participants’ ‘views and opinions of situations, corresponding patterns, themes, categories and regular similarities’ (Cohen, Manion & Morrison 2007:461). The data, which were collected in the form of field notes and through audio recording, were transcribed and translated. The field notes served as backup to the data collected through audio recording. Data were then translated from isiNdebele language of the research participants, to English for the study.

### Ethical considerations

As qualitative research entails entering into the personal domains of research participants, it was prudent for the authors to follow research ethics in order to increase response rate. The authors considered, among other issues, issues of informed consent, confidentiality and anonymity of the participants. All the participants were volunteers who were free to withdraw at any stage they wished. They were also assured of utmost confidentiality of their data and of their identities. According to Chaminuka and Dube (2017), ethical considerations can promote the accomplishment of study aims, as well as promote cooperation from participants. Furthermore, the authors assured of no physical or psychological harm to be suffered by the participants.

## Results and discussion

This section presents and discusses the results of the study. The presentation of the results is in line with the themes. The themes were chosen so that the findings of the study are in line with the research objectives.

### Impact of flood disasters on the built environment in Tsholotsho district

This study revealed that floods in Tsholotsho district impacted the built environment through damaging human shelter or dwellings, roads, bridges and dams (Table 1). From the respondents’ narrations, it was discovered that the type of human shelter damaged by the floods consisted mainly of huts that were made of pole and mud.

**TABLE 1:** Type of structures impacted by floods in the built environment (*N* = 40).

Type of structure	Nature of flood impact/damage	Number of respondents	Percentage of respondents
Human shelter	Huts made of pole, mud and thatch collapsed after absorbing water Some huts in low-lying areas were submerged in water	20	50.0
Roads	Roads eroded by runoff, resulting in potholes Some roads were completely submerged in water and rendered unusable	10	25.0
Bridges	Bridges were submerged in water, becoming impassable Some bridges were broken and carried away by heavy currents, and road network was disconnected	05	12.5
Dams	Dam walls were washed away by heavy currents of water	05	12.5

**Total**		**40**	**100.0**

Table 1 reflects the different views of the 40 respondents on the type of structures mostly affected by the floods in the district. According to the respondents, the structures mostly affected included human shelter, roads, bridges and dams. These structures represent the physical capital that the flood affected communities depended on for their livelihoods. Twenty out of the 40 respondents (50.0%) mentioned that human shelter, which was made of mud, pole and thatch, was at the forefront of the structures heavily impacted, followed by roads (10 respondents – 25.0%), bridges (5 respondents – 12.5%) and dams (5 respondents – 12.5%). According to the respondents, the damaged roads consisted of a tarred road linking Tsholotsho district with Lupane district to the north, and the city of Bulawayo to the west, and one gravel road linking Tsholotsho district with Butabubili area to the east. The following excerpt encapsulates a lived-experience:

‘The 2012 flood was so severe that it destroyed bridges and dam walls. A bridge was damaged along Zombani River, and a dam wall was destroyed at Siphepha dam. When Zombani River is flooded, we are not able to cross safely’. (Male villager, 54 years, Tamuhla village, Siphepha area)

The findings in Table 1 were further corroborated by observations made by the researchers. The observation method was meant to provide first-hand information about the structures impacted by the floods in the built environment. The researchers observed that huts made of pole, mud and thatch were destroyed. The infrastructure that included poorly constructed bridges and dam walls were either partially damaged or completely destroyed. These findings agree with results from previous studies by Mudavanhu et al. (2015) in Muzarabani, who established that floods resulted in the collapse of houses, destruction of bridges and roads were rendered unusable. The findings further corroborate a study by Munich Re (2013:63) in Bangladesh, which showed that the 2012 Bangladesh floods destroyed over 250 000 housing units. Following the interpretive paradigm of this study, it could be interpreted that by destroying the infrastructure, floods are a selective and powerful phenomenon and a threat to the most vulnerable members of societies, and to their assets.

### Factors that perpetuate flood vulnerability in the built environment

Three major factors were found to have contributed to and perpetuated flood vulnerability, and flood disasters in the built environment of Tsholotsho district. According to the interviewees, such factors include location of homesteads near rivers, dams and other water basins – which was most mentioned (25.0% – 62.5%), followed by use of non-durable or poor-quality construction materials (10.0%– 25.0%) and lack of timely flood warning (5.0% – 12.5%). These factors are discussed in detail in the subsection ‘Homesteads located near rivers, dams and other water basins’.

#### Homesteads located near rivers, dams and other water basins

Homesteads located near rivers and dams proved to be the major factor perpetuating flood vulnerability in the district. Of the interviewed flood victims, 62.5% were of the view that had their settlements been sited away from the rivers and dams, they would have been free from flood risk. This can be interpreted to mean that the communities settled in flood-prone areas were aware of the dangers facing them, but just ignored them. As such, disasters can impact even those communities with enough disaster knowledge and awareness if no proper measures are taken. Through the researchers’ observations, it was also noted that some villagers in Wards 5 and 6 were located very close to the Zombani and Gwayi rivers respectively, while those in Ward 8 were situated close to Gariya and Bhudani dams. One villager narrated how during the rainy season, water from the Bhudani dam would overflow into their homesteads:

‘During rainy seasons, water overflows to our homesteads when Bhudani dam is full. This situation has resulted in the community losing some property in the form of huts, barns and school buildings in addition to the destroyed dam wall’. (Female villager, 36 years, Bhudani area)

The above findings concur with a study conducted by Parker, Little and Heuser (2007), who found that growing populations in areas at risk of flooding contributed to disasters. The findings are also linked to the social constructionist theory. Social constructionists are asking for conditions that cause vulnerability. They view climate events such as flooding as external phenomena, and they place the burden of explanation of vulnerability within the social system that humans create (Ribot 2009). The communities should desist from the practice of settling in the proximity of rivers and dams. In this way, the impact of flood disasters on the built environment could be ameliorated. The authors, therefore, argue that the impacts of flood disasters in Tsholotsho district are a social construct, instigated and amplified by human actions.

#### Use of non-durable construction material

Apart from building their homesteads in flood-prone areas and near rivers and dams, communities were observed to have used non-durable materials for building their structures. Some respondents (10.0% – 25.0%) thought their flood vulnerability was worsened by weak materials used in the construction of shelter (huts and houses), roads, bridges and dam walls. Dwelling structures, according to the respondents and complemented by observations, were constructed from pole, mud and thatch. This type of building material was observed to be inferior and prone to flood damage. The continued use of such material in Tsholotsho district suggests that the district might continue to be a breeding place for flood disasters. Poor workmanship in the construction of bridges was observed by the researchers.

One such bridge which was observed to have been damaged by the floods was a small bridge crossing Zombani River in Siphepha area. The bridge was completely washed away by flood waters, rendering it dangerous and unusable. Crossing places along rivers need to be constructed using durable material that does not endanger the lives of the rural communities. The findings are in line with results from Sharma and Joshi (2008), who found that structures made of weak material, such as mud, cannot resist water, thereby rendering them prone to severe flood damage. It is logical that structures constructed in flood-prone areas are made of material that can withstand forces from flood waters. Lack of precautionary measures on the damaged infrastructure shows that the communities were not anticipating the damage. They might have underrated the flood impact. Otherwise, more durable and stronger material was supposed to be used for construction as a form of flood preparedness and mitigation. According to the UN-Habitat (2012), most of the housing structures damaged by the 2010 floods in Pakistan, were made of mud and adobe brick, while houses built from concrete and burnt brick survived the flood impact because they were more resistant.

#### Lack of flood awareness

Lack of flood awareness within the communities was found to be another factor contributing to flood vulnerability in Tsholotsho district. Some respondents (5.0% – 12.5%) highlighted that most people lacked sufficient information on impending flood hazards, resulting in being caught unawares. The respondents were of the opinion that lack of flood awareness contributed to their ignorance about flood hazards, hence their increased vulnerability to flooding. Their argument was that communities with enough flood awareness would rarely be caught unaware during periods of severe flooding. Respondents felt that flood awareness programmes should be undertaken within the communities, and that these programmes would make known flood hazards existing within their vulnerability contexts. One respondent had this to say:

‘We want the responsible authorities, especially the District Civil Protection Unit, to come down here to the communities and provide flood awareness programmes. People living in this part of the district do not have enough information concerning their vulnerabilities to flooding, hence our continued suffering’. (Male villager, 34 years, Mahlosi village)

However, the study further established from the respondents that awareness campaigns had been provided by the Tsholotsho District Civil Protection Unit (DCPU), although they have been few. From the respondents’ narrations, provision of timely flood awareness by the DCPU may enhance their capacity to prepare for flooding events in the district. As such, this study interprets that the DCPU and its partners are not effective enough to serve the communities well. Disaster knowledge is an empowerment tool against flooding, and communities should possess such knowledge in advance and in abundance (Fabiyi & Oloukoi 2013; Madhuri et al. 2015). Communities with disaster knowledge are thus empowered to avoid disaster losses. These findings concur with a study by Bhaduri (2013), who found that the state and co-operating partners can enhance preparedness to disasters through outreach programmes and provision of awareness in communities. Provision of sufficient and timely flood awareness information by the duty-bearers is, therefore, what the communities need to properly act against flood hazards.

### Challenges that can negate the management of flood disasters in the built environment

Three major issues were found to negate the management of flood disasters in the built environment of Tsholotsho district. As highlighted by 20 out of 40 (50.0%) respondents, poverty and cost of rebuilding infrastructure, followed by lack of cooperation between communities and duty-bearers (15.0% – 37.5%), and failure to properly use indigenous knowledge (5.0% – 12.5%) were found to be the main challenges affecting the rebuilding processes.

#### Poverty and costs of rebuilding infrastructure

**FIGURE 2:**
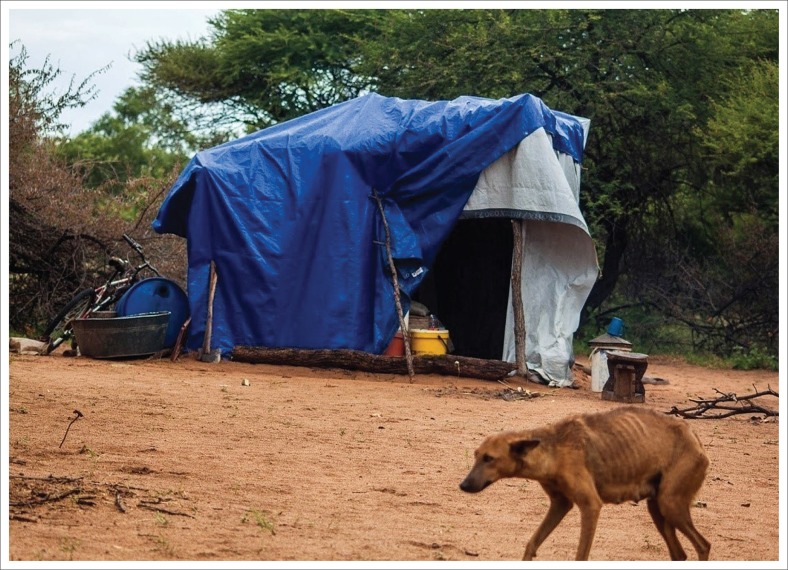
A tent that has been converted into a permanent dwelling at Butabubili village.

The majority of people in places affected by the floods in Tsholotsho district, as highlighted by the respondents, are peasant farmers, most of whom are languishing in poverty. This study views poverty as being based on the evaluation of the economic or income status of individual members of the community (Ayala, Jurado & Perez-Mayo 2009). According to the respondents, people affected by the floods in the district were found to have limited sources of income to finance the rebuilding process in the aftermath of destructive flooding events. As such, this study views poverty as a development barrier, owing to the fact that its high levels can increase vulnerability and retard the pace of development. Respondents stated that their poverty status forced them to either rebuild similar inferior structures after flooding or to convert temporary shelter constructed from zinc sheets, wooden poles or tents, into permanent accommodation. This indicates that the poor have limited choices and are forced to live in any type of shelter. According to Mtapuri (2008:38), the poor are also those who live in ‘poor dwellings’. The respondents further indicated that they cannot afford the cost of purchasing suitable building materials to construct decent houses. To complement the narrations made by the respondents, the researchers observed that one family at Butabubili village had converted a donated tent meant to be used as temporary shelter, into a permanent dwelling (Figure 2). The structure does not add value to the built environment, as it perpetuates vulnerability to future flood hazards. These findings complement a study by Save the Children (2006:6) carried out in Bangladesh, which found that ‘poverty is intrinsically linked with the impact that floods have on any given segment of the population…’

Figure 2 depicts a donated tent for use by one family on a temporary basis, while suitable accommodation was being sought. However, according to one respondent, the tent had been in use for more than a year, effectively turning it into a permanent dwelling. Duty-bearers have an obligation to ensure that shelter provided on a temporary basis is not used for a long period.

#### Lack of cooperation between members of the community and duty-bearers

Inability by both members of the community and the duty-bearers to cooperate in disaster risk reduction programmes was another challenge affecting the management of flood disasters. As noted by the respondents, some members of the community resisted the government idea to be relocated to safer areas, where they were promised better houses. The respondents further narrated that their resistance emanated from the government’s decision to relocate them without consulting them. Hence, the resistance resulted in lack of cooperation between the duty-bearers and the communities. As a result, organisations willing to provide aid in the rebuilding phase could not offer it unless the affected populations had relocated. One villager stated:

‘Flood victims do not trust the government and humanitarian agencies. They feel that the promise by the government and its partners to build better houses for them does not hold water, as they view it as a way to simply successfully relocate them from their current places. As such, the communities have refused to cooperate with the authorities’. (Female villager, 34 years, Gariya area)

People expect the responsible governments to help them to recover from disasters. Therefore, the government as the main duty-bearer should not impose humanitarian aid on the communities without proper consultation. These results support findings from previous research found in the extant literature. For example, Cannon, Twigg and Rodwell (2003:10) showed that communities and organisations that have cooperated successfully were in a position to assist each other and other groups during disaster events. Lack of cooperation between the communities and the duty-bearers can result in a situation where flood-impacted communities find themselves in worse conditions.

#### Failure by the duty-bearers to consider indigenous knowledge

Members of the community living with flood risk were found to command indigenous knowledge for dealing with floods. According to the respondents, they were able to use home-grown knowledge to forecast rainy seasons with a potential to cause flooding. This guided them in taking appropriate action in preparation for flooding. The respondents narrated how they studied and interpreted the behaviour of certain animals and birds to forecast the magnitude of rains. They studied the behaviour of *inkanku* (a bird associated with forecasting rains), cloud patterns and changes in certain indigenous trees. The respondents believed such indigenous knowledge helps them to predict rains with a potential for flooding. From the researchers’ interpretive point of view, the use of indigenous knowledge by the residents is symbolic of the norms, culture and beliefs prevailing in many African communities. It also shows the value that African communities attach to their cultural beliefs and how their culture shapes their daily lives. These findings are consistent with the results of a study by Domfeh (2007) on indigenous knowledge systems in Swaziland, who discovered that the availability of specific types of birds on trees was a sign of the beginning of the rainy season, and that flood forecasting is also associated with how high birds build their nests from river surfaces.

Although the knowledge generated from the local communities is of utmost relevance, the respondents felt that their knowledge was underrated by the DCPU and other players. The respondents thought that local communities should be allowed to contribute their knowledge in managing flood disasters. The authors interpret this as low-rating of indigenous knowledge compared to Western knowledge. Again, disaster risk reduction practitioners see no potential value of indigenous knowledge as an effective tool for flood disaster management. However, the respondents were of the opinion that their indigenous knowledge should be integrated with modern knowledge, so that both spheres of knowledge become more effective. Their argument was that as the practitioners fall short of using indigenous knowledge that communities possess, infusing the two types would be a positive step.

## Recommendations

Drawing from the findings and conclusions, the authors recommend duty-bearers to assist flood-impacted communities with the means of restoring their built environments. Duty-bearers should assist communities with material for rebuilding of flood-resistant structures. In addition, regular flood awareness is to be undertaken to alert the communities in flood-prone areas. Lastly, duty-bearers should consider integrating indigenous knowledge of the communities with modern knowledge in disaster issues.

## Conclusion

This article concludes that flood disasters can impact the built environment through damaging infrastructure. Therefore, flood disasters have a propensity to wipe out community development gains accumulated over many years. The article further concludes that perpetuation of flood vulnerability in the built environment is an avoidable social construct created through people’s interaction with their environment. This study views the build-back-better concept as viable option to development in the aftermath of flood damage. However, challenges exist that may impede the management of flood disasters affecting the built environment. Finally, the study concludes that reconstruction following flood disasters is difficult and arduous.

## References

[CIT0001] ANHLC (Association of Neighbourhood Houses & Learning Centres), (n.d), *Community Development Principles*, viewed 13 September 2017, from https://www.google.co.za/search?q=prpinciples+of+community+development&rlz=1C1KYPA_enZA648ZA678&oq=prpinciples+of+community+development&aqs=chrome.69i57j0l5.7400j0j8&sourceid=chrome&ie=UTF-8

[CIT0002] Ariyabandu M.M. & Wickramasinghe M., 2003, *Gender dimensions in disaster management*, ITDG South Asia Publication, Colombo.

[CIT0003] Ayala L., Jurado A. & Perez-Mayo J., 2009, *Income poverty and multidimensional deprivation: Lessons from cross-regional analysis*, ECINEQ Working Paper No. 106. Society for the Study of Income Inequality, Palma de Mallorca.

[CIT0004] Baradhan B., 2006, ‘Analysis of the post-disaster reconstruction process following Turkish Earthquakes, 1999’, in IF Research Group (ed.), International conference on post-disaster reconstruction meeting stakeholder interests, May 17–19, 2006, University de Montreal, Florence, pp. 1–14.

[CIT0005] Barenstein J.D. & Pittet D., 2007, ‘Post-disaster housing reconstruction current trends and sustainable alternatives for tsunami-affected communities in coastal Tamil Nadu’, Institute for Applied Sustainability to the Built Environment, The University of Applied Sciences of Southern Switzerland.

[CIT0006] Benson C. & Clay E., 2004, *Understanding the economic and financial impact of natural disasters*, World Bank, Washington, DC.

[CIT0007] Bhaduri A., 2013, ‘Impact of climate change on life & livelihood of Dalits: An exploratory study from disaster risk reduction lens’, NCDHR & SPWD, New Delhi.

[CIT0008] Birkland T.A., 2006, *Lessons of disaster: Policy change after catastrophic events*, Georgetown University Press, Washington, DC.

[CIT0009] Boen T. & Jigyasu R., 2005, *Cultural considerations for post disaster reconstruction post tsunami challenges*, Bangkok, Asian Disaster Preparedness Centre.

[CIT0010] Cannon T., Twigg J. & Rodwell J., 2013, *Social vulnerability, sustainable livelihoods and disasters*, Report to DFID, Natural Resources Institute, London.

[CIT0011] Chaminuka N. & Dube E., 2017, ‘Urban agriculture as a food security strategy for urban dwellers: A case study of Mkoba residents in the city of Gweru, Zimbabwe’, *PEOPLE: International Journal of Social Sciences* 3(2), 26–45. https://doi.org/10.20319/pijss.2017.32.2645

[CIT0012] Cigler B.A., 2017, ‘U.S. floods: The necessity of mitigation’, *State and Local Government Review* XX(X), 1–13. https://doi.org/10.1177/0160323X17731890

[CIT0013] Cohen L., Manion L. & Morrison K., 2007, ‘Research methods in education’, *British Journal of Education Studies* 55(4), 1–638.

[CIT0014] Collins E. & Simpson L., 2007, *The impact of climate change on insuring flood risk*, Institute of Actuaries of Australia, New Zealand, pp. 1–38.

[CIT0015] Da Silva J., 2009, *Lessons from Aceh: Key considerations in post-disaster reconstruction*, Practical Action Publishing, Warwickshire.

[CIT0016] Davidson C.H., Johnson C., Lizarralde G., Dikmen N. & Sliwinski A., 2007, ‘Truths and myths about community participation in post-disaster housing projects’, *Habitat International* 31(1), 100–115. https://doi.org/10.1016/j.habitatint.2006.08.003

[CIT0017] Davis I., 2005, *What makes a disaster?* viewed 27 March 2016, from http://tilz.tearfund.org/Publications/Footsteps+11-20/Footsteps+18

[CIT0018] Delaney P.L. & Shrader E., 2000, *Gender and post-disaster reconstruction: The case of Hurricane Mitch in Honduras and Nicaragua*, Report prepared for the World Bank, viewed 08 May 2016, from www.anglia.ac.uk/geography/gdn

[CIT0019] Domfeh K.A., 2007, ‘Indigenous knowledge systems and the need for policy and institutional reforms. Tribes and tribals, indigenous knowledge systems and sustainable development’, *Relevance for Africa* 1(5), 41–52.

[CIT0020] Dube E., 2015, ‘Improving disaster risk reduction capacity of District Civil Protection Units in managing veld fires: A case of Mangwe District in Matabeleland South Province, Zimbabwe’, *Jàmbá: Journal of Disaster Risk Studies* 7(1), 1–13. https://doi.org/10.4102/jamba.v7i1.143 10.4102/jamba.v7i1.143 PMC6013976 29955275

[CIT0021] Dube E., 2017, ‘Towards enhanced disaster risk management interventions for flood hazards and disasters in Tsholotsho District, Zimbabwe’, Unpublished PhD thesis, Midlands State University (MSU), Gweru.

[CIT0022] Environmental Planning Collaborative [EPC] and TCG International, 2004, *Participatory planning guide for post-disaster reconstruction*, USAID/India and the Indo-US Financial Institutions Reform and Expansion (FIRE-D), Ahmadabad.

[CIT0023] Fabiyi O.O. & Oloukoi J., 2013, ‘Indigenous knowledge system and local adaptation strategies to flooding in Coastal Rural Communities of Nigeria’, *Journal of Indigenous Social Development* 2(1), 1–19.

[CIT0024] Fu T.H., Lin W.I. & Shieh J.C., 2013, ‘The impact of post-disaster relocation on community solidarity: The case of post-disaster reconstruction after Typhoon Morakot in Taiwan’, World Academy of Science, Engineering and Technology, *International Science Index* 7(6), 1978–1981.

[CIT0025] Google images, 2013, *Map of Tsholotsho District, Zimbabwe*, viewed 10 August 2017, from http://images.google.com/images?hl=EN&biw=1366&bih=677&gbv=2&tbs=isch:1&sa=1&q=map+of+tsholotsho+district+in+zimbabwe&aq=f&aqi=&aql=&oq=&gs_rfai=

[CIT0026] Harvey P., 2005, *Cash and vouchers in emergencies*, HPG Discussion Paper, Humanitarian Policy Group at Overseas Development Institute, London.

[CIT0027] Henderson L.J., 2004, ‘Emergency and disaster: Pervasive risk and public bureaucracy in developing nations. Public Organization Review’, *A Global Journal* 4, 103–119.

[CIT0028] Hewitt K., 1983, ‘The idea of calamity in a technocratic age’, in Hewitt K. (ed.), *Interpretations of Calamity: From the viewpoint of human ecology*, pp. 3–32, Allen & Unwin, Boston, MA.

[CIT0029] Johnson C., Lizarralde G. & Davidson C.H., 2006, ‘A systems view of temporary housing projects in post-disaster reconstruction’, *Construction Management & Economics* 24, 367–378. https://doi.org/10.1080/01446190600567977

[CIT0030] Lindell M.K., 2013, *Recovery and reconstruction after disaster*, Federal Emergency Management Agency Emergency Management Institute, Emmitsburg, MD, viewed 06 October 2016, from archone.tamu.edu/hrrc/Publications/books/index.html

[CIT0031] Lo D.S. & Oreta W.C., 2010, ‘Seismic risk mapping at micro-scale: The case of Barangay Carmen, Cagayan de Oro City, Philippines’, Conference on ‘Harnessing Lessons Towards an Earthquake-Resilient Nation’, PhiVolcs, Quezon City, July 15–16, 2010, pp. 1–10.

[CIT0032] Madhuri, Tewari H.R., Bhowmick P.K. & McCormick M., 2015, ‘Roles of government and community support, flood experience, and flood education in livelihood resilience’, *Journal of Sociology & Social Welfare* XLII(4), 101–133.

[CIT0033] Mannakkara S., Wilkinson S. & Potangaroa R., 2014, ‘Build-back-better: Implementation in Victorian bushfire reconstruction’, *Disasters* 38(2), 267–290. https://doi.org/10.1111/disa.12041 24601917 10.1111/disa.12041

[CIT0034] Manyena S.B., 2009, ‘Disaster resilience in development and humanitarian intervention’, PhD thesis, School of Applied Science, Northumbria University, Newcastle.

[CIT0035] McEntire D.A., 2003, ‘Causation of catastrophe: Lesson from Hurricane George’, *Journal of Emergency Management* 1(2), 22–29.

[CIT0036] McEntire D.A., 2004, ‘Development, disasters and vulnerability’, *Disaster Prevention and Management* 13(3), 193–198. https://doi.org/10.1108/09653560410541786

[CIT0037] Mileti D.S., Darlington J.D., Passarini E., Forest B.C. & Myers M.F., 1995, ‘Toward an integration of natural hazards and sustainability’, *Environmental Professional* 17(2), 117–126.

[CIT0038] Mtapuri O., 2008, ‘Exploring local conceptions of poverty, wealth and well-being: Field evidence from Mashonaland West Province of Zimbabwe’, *Africa Development* 3, 35–54.

[CIT0039] Mudavanhu C., 2014, ‘The impact of flood disasters on child education in Muzarabani District, Zimbabwe’, *Jàmbá: Journal of Disaster Risk Studies* 6(1), Art. #138, 8 pages. https://doi.org/10.4102/jamba.v6i1.138

[CIT0040] Mudavanhu C., Manyena B.S., Collins A.E., Bongo P.P., Mavhura E. & Manatsa D., 2015, ‘Taking children’s voices in disaster risk reduction a step forward’, *International Journal of Disaster Risk Science* 6(3), 267–281. https://doi.org/10.1007/s13753-015-0060-7

[CIT0041] Munich Re., 2013, *Topics geo: Natural catastrophes 2012: Analyses, assessments, positions*, Munich Re Group, Munich.

[CIT0042] Oelofse C., 2002, ‘Dimensions of urban environmental risk’, in Nomdo & Coetzee C. (eds.), *Urban vulnerability: Perspectives from Southern Africa*, pp. 28–53, Peri Peri Publications, Cape Town.

[CIT0043] Ozden A.T., 2004, ‘Evaluation of post-disaster housing in Senirkent’, Unpublished Master thesis, Institute of Natural and Applied Sciences, Istanbul Technical University, Istanbul.

[CIT0044] Parker R., Little K. & Heuser S., 2007, *Development actions and the rising incidence of disasters (Evaluation Brief 4)*, World Bank, Washington, DC.

[CIT0045] Patton M.Q., 2002, *Qualitative research and evaluation methods*, Sage, Thousand Oaks, CA.

[CIT0046] Pelling M., 2003, *The vulnerability of cities: Natural disasters and social resilience*, Earthscan, London.

[CIT0047] Price R.K. & Vojinovic Z., 2008, ‘Urban flood disaster management’, *Urban Water Journal* 5(3), 259–276. https://doi.org/10.1080/15730620802099721

[CIT0048] Ribot J.C., 2009, ‘Vulnerability does not just fall from the sky: Toward multi-scale pro-poor climate policy’, in Mearns R. & Norton A. (eds.), *Social dimensions of climate change: Equity and vulnerability in a warming world*, pp. 1–21, The World Bank, Washington, DC.

[CIT0049] Satterthwaite D., Saleemul H., Pelling M., Reid H. & Lankao P.R., 2007, *Adapting to climate change in urban areas: The possibilities and constraints in low- and middle income nations*, Human Settlements Discussion Paper Series, International Institute for Environment and Development (IIED), London.

[CIT0050] Save the Children, 2006, *Watermarks: Child protection during floods in Bangladesh*, Save the Children, Dhaka.

[CIT0051] Schilderman T., 2004, ‘Adapting traditional shelter for disaster mitigation and reconstruction: Experiences with community-based approaches’, *Building Research and Information* 32(5), 414–426. https://doi.org/10.1080/0961321042000250979

[CIT0052] Shah A., Khan H.M. & Qazi E.U., 2013, ‘Damage assessment of flood affected mud houses in Pakistan’, *Journal of Himalayan Earth Sciences* 46(1), 99–110.

[CIT0053] Sharma A. & Joshi M., 2008, ‘Indigenous knowledge and modern science give environment friendly shelter solution in flood affected Desert Region of India’, in Shaw R., Uy N. & Baumwo J. (eds.), *Indigenous knowledge for disaster risk reduction: Good practices and lessons learned from experiences in the Asia-Pacific Region*, pp. 9–13, UNISDR, Bangkok.

[CIT0054] Steinberg F., 2007, ‘Housing reconstruction and rehabilitation in Aceh and Nias, Indonesia –Rebuilding lives’, *Habitat International* 31(1), 150–166. https://doi.org/10.1016/j.habitatint.2006.11.002

[CIT0055] Stephenson R.S., 1994, *Disasters and development*, UNDP, DHA, New York.

[CIT0056] Thurairajah N., Amaratunga D. & Haigh R., 2008, ‘Post-disaster reconstruction as an opportunity for development: Women’s perspective’, CIB W89: International conference in building education and research, February 10–15, 2008, pp. 1106–1115, Kandalama, Sri Lanka.

[CIT0057] UN-Habitat, 2012, *Pakistan Settlements Flood recovery programme (PSFRP) – Project completion report December 2012*, UN, Fukuoka.

[CIT0058] Velasquez G. & Tanhueco R.M.T., 2005, ‘Incorporating social issues in disaster risk assessment’, in Velasquez G. & Tanhueco R.M.T. (eds.), *Know risk*, pp. 91–92, United Nations, Geneva.

[CIT0059] Zimstat, 2012, *Census 2012 National Report*, viewed 03 October 2017, from http://www.zimstat.co.zw/documents/census

[CIT0060] Zuo K. & Wilkinson S., 2008, ‘Supply chain and material procurement for post disaster construction: The Boxing Day Tsunami reconstruction experience in Aceh, Indonesia’, CIB W89 International Conference on Building Education and Research BEAR 2008, February 11–15, 2008, Heritance Kandalama, Sri Lanka, pp. 1116–1133.

